# Certain Actions from the Functional Movement Screen Do Not Provide an Indication of Dynamic Stability

**DOI:** 10.1515/hukin-2015-0058

**Published:** 2015-10-14

**Authors:** Robert G. Lockie, Samuel J. Callaghan, Corrin A. Jordan, Tawni M. Luczo, Matthew D. Jeffriess, Farzad Jalilvand, Adrian B. Schultz

**Affiliations:** 1 Department of Kinesiology, California State University, Northridge, Northridge, USA.; 2 School of Exercise and Health Sciences, Edith Cowan University, Joondalup, Australia.; 3 Exercise and Sport Science, School of Environmental and Life Sciences, University of Newcastle, Ourimbah, Australia.; 4 Department of Kinesiology, California State University of Monterey Bay, Seaside, USA.; 5 Faculty of Health, University of Technology, Sydney, Lindfield, Australia.

**Keywords:** Star Excursion Balance Test, functional reaching, screening, in-line lunge, trunk stability push-up

## Abstract

Dynamic stability is an essential physical component for team sport athletes. Certain Functional Movement Screen (FMS) exercises (deep squat; left- and right-leg hurdle step; left- and right-leg in-line lunge [ILL]; left- and right-leg active straight-leg raise; and trunk stability push-up [TSPU]) have been suggested as providing an indication of dynamic stability. No research has investigated relationships between these screens and an established test of dynamic stability such as the modified Star Excursion Balance Test (mSEBT), which measures lower-limb reach distance in posteromedial, medial, and anteromedial directions, in team sport athletes. Forty-one male and female team sport athletes completed the screens and the mSEBT. Participants were split into high-, intermediate-, and low-performing groups according to the mean of the excursions when both the left and right legs were used for the mSEBT stance. Any between-group differences in the screens and mSEBT were determined via a one-way analysis of variance with Bonferroni post hoc adjustment (p < 0.05). Data was pooled for a correlation analysis (p < 0.05). There were no between-group differences in any of the screens, and only two positive correlations between the screens and the mSEBT (TSPU and right stance leg posteromedial excursion, r = 0.37; left-leg ILL and left stance leg posteromedial excursion, r = 0.46). The mSEBT clearly indicated participants with different dynamic stability capabilities. In contrast to the mSEBT, the selected FMS exercises investigated in this study have a limited capacity to identify dynamic stability in team sport athletes.

## Introduction

The Functional Movement Screen (FMS) is often used to monitor functional capacity, as the actions have been described as challenging an individual’s ability to expedite movement in a proximal-to-distal fashion (Cook et al., 2006a). Traditionally, the FMS has been used as a potential indicator of injury risk in athletes (Chorba et al., 2010; Kiesel et al., 2007), although further research is needed to confirm this relationship (Teyhen et al., 2014). More recently, the FMS has been investigated with regard to its relationship to athletic performance (Lockie et al., 2013a; Lockie et al., 2015; Parchmann and McBride, 2011), given that effective movement patterns are needed for sport.

However, research has found limitations with the FMS in providing an indication of ineffective movement patterns that influence athletic performance. For example, multidirectional speed has been found to have minimal relationships with the FMS, including 20 m sprint and T-test performance in collegiate golfers (Parchmann and McBride, 2011), and 20 m sprint, 505 change-of-direction speed test, and modified T-test performance in male team sport athletes (Lockie et al., 2015). Nonetheless, it should be noted that multidirectional speed incorporates a number of physical capacities, one of which includes dynamic stability (Sheppard and Young, 2006). In recent times, this capacity has been investigated in team sport athletes (Lockie et al., 2013b; Lockie et al., 2014b, in press; Thorpe and Ebersole, 2008).

Within multidirectional movements, athletes must maintain stability when transitioning from a dynamic (deceleration) to a static (stopping in preparation to change direction), before returning to a dynamic (re-acceleration) state. A valid and popular assessment of dynamic stability is the Star Excursion Balance Test (SEBT), which utilizes functional reaching of the legs from a unilateral stance in eight directions (anterior, anterolateral, lateral, posterolateral, posterior, posteromedial, medial, and anteromedial) (Olmsted et al., 2002; Robinson and Gribble, 2008). The SEBT is a valuable test, as it may predict the risk of leg injuries in athletes (Dallinga et al., 2012; Plisky et al., 2006), while more importantly for this study, also relates to athletic performance (Lockie et al., in press; Thorpe and Ebersole, 2008). When compared to non-athletes, collegiate female soccer players could reach further in anterior and posterior directions (Thorpe and Ebersole, 2008). Lockie et al. (in press) found that faster male team sport athletes in assessments such as the 40 m sprint, T-test, and change-of-direction and acceleration tests, could reach further in the medial and posteromedial directions.

Given the importance of dynamic stability for team sport athletes (Lockie et al., 2014b, in press; Sheppard and Young, 2006), there is value for strength and conditioning coaches to understand whether other tests also provide an indication of this physical quality, and potentially identify physical deficiencies affecting performance. Although the FMS has been found not to relate to multidirectional sprinting itself (Lockie et al., 2015; Parchmann and McBride, 2011), screens that require a stable base during movement may be able to provide an indication of a component of speed in dynamic stability. In addition to this, FMS literature has implied the importance of dynamic stability to the screening movements (Cook et al., 2006a, 2006b). Indeed, Teyhen et al. (2014) found small-to-moderate correlations between the Y-balance test and the deep squat (correlation and coefficient [r] = 0.38), hurdle step (r = 0.34), and in-line lunge (r = 0.40), in male and female active duty service members. Research investigating relationships between the FMS and an established test of dynamic stability specific to team sport athletes could provide strength and conditioning coaches the opportunity to use certain screening exercises as a means to identifying movement limitations affecting this capacity. This would also confirm whether anecdotal recommendations as to the importance of dynamic stability within screening exercises are appropriate.

Therefore, this study analyzed the relationship between individual FMS assessments (a deep squat, a hurdle step, an in-line lunge, an active straight-leg raise, and a trunk stability push-up) with performance in a modified SEBT (mSEBT) in team sport athletes. The mSEBT utilizes only the posteromedial, medial, and anteromedial excursions, and eliminates redundant measurements to make the assessment more efficient (Hertel et al., 2006). Participants were split into high-, intermediate-, and low-performing groups according to the mean of reach scores attained for each leg when used for the stance in the mSEBT. This demonstrated whether athletes who had better dynamic stability were superior in the selected screens from the FMS. As these screens had been said to require some form of dynamic stability and movement control (Cook et al., 2006a, 2006b), it was hypothesized that participants who demonstrated superior dynamic stability would also perform better in these screens. Additionally, higher scores in the hurdle step and the in-line lunge would correlate with further excursion distances.

## Material and Methods

### Participants

Forty-one recreational team sport athletes (age = 22.80 ± 4.13 years; body height = 1.76 ± 0.09 m; body mass = 76.05 ± 12.85 kg), including 32 males (age = 22.84 ± 3.90 years; body height = 1.79 ± 0.07 m; body mass = 79.37 ± 12.49 kg) and 9 females (age = 22.67 ± 5.12 years; body height = 1.66 ± 0.05 m; body mass = 64.22 ± 4.44 kg), volunteered for this study. Mixed-gender groups have been previously used in the FMS (Okada et al., 2011; Parchmann and McBride, 2011; Teyhen et al., 2014), and sport (Eikenberry et al., 2008; Guissard et al., 1992; Lockie et al., 2012; Spiteri et al., 2013) research. Participants were recruited if they: currently played a team sport (soccer, netball, basketball, rugby, Australian football, touch football); were currently training for a team sport (≥three times per week); and had a training history (≥two times per week) extending over the previous year. Although there may be certain differences in traits between different sport participants, the analysis of performance with regard to physical characteristics common to athletes from assorted team sports had been consistently conducted within the literature (Lockie et al., 2014a; Lockie et al., 2011; Sassi et al., 2009; Sekulic et al., 2013; Spiteri et al., 2013). To limit the influence of any injuries that could affect FMS scoring, participants were only included if they had not sustained an injury in the previous 30 days that prohibited them from full participation in regular training and competition (Chorba et al., 2010). The study occurred within the competition season for all participants, and the procedures were approved by the University of Newcastle ethics committee. All subjects received a clear explanation of the study, including the risks and benefits of participation, and written informed consent was obtained prior to testing.

### Procedures

Data was collected over two sessions, separated by one week. The first session involved the FMS assessments, while the second testing session incorporated the mSEBT. Prior to the FMS assessment in the first session, each participant’s age, body height, and body mass were recorded. Body height was measured using a stadiometer (Ecomed Trading, Seven Hills, Australia), while body mass was recorded using electronic digital scales (Tanita Corporation, Tokyo, Japan). Participants then completed the selected screens. In the second session, the mSEBT warm-up consisted of low-intensity cycling on a bicycle ergometer, followed by circuits of the mSEBT, the specifics of which will be documented. Participants were tested at the same time of day for both sessions and in the same order, did not eat for 2–3 hours prior to their testing sessions, and refrained from taking any stimulants such as caffeine, or intensive lower-body exercise, in the 24 hours prior to testing.

### Functional Movement Screen (FMS)

Five movements were used from the FMS for this study, and the intra-rater reliability of these screens had been previously established (Minick et al., 2010; Onate et al., 2012). Although Shultz et al. (2013) documented some limitations in the inter-rater reliability of the FMS, as will be detailed, the procedures adopted in this study sought to limit the influence of this. The selected screening tests, as described by Frost et al. (2012), were completed in the following order: 1. deep squat: a dowel was held overhead with arms extended, and the participant squatted as low as possible; 2. hurdle step: a dowel was held across the shoulders, and the participant stepped over a hurdle in front of them that was level with their tibial tuberosity; 3. in-line lunge: with a dowel held vertically behind the participant such that it contacted the head, back and sacrum, and with the feet aligned, the participant performed a split squat; 4. straight-leg raise: lying supine with their head on the ground, the participant actively raised one leg as high as possible; and 5. trunk stability push-up: the participant performed a push-up with their hands shoulder-width apart. As stated, these screens were selected as they had been said to require some form of dynamic stability (Cook et al., 2006a, 2006b). The shoulder mobility test was not used as it consists of completely isolated movement to the glenohumeral joint (Cook et al., 2006b). The rotary stability test was excluded because previous research had stated that it was not a practical test for athletic populations (Schneiders et al., 2011). A clearing test was employed for the trunk stability push-up, where the participant performed a press-up from the push-up start position, while maintaining contact between the hips and the ground (Cook et al., 2006b).

FMS scoring checklists had been presented in the literature (Cook et al., 2006a, 2006b; Frost et al., 2012; Okada et al., 2011), and were used for this study. Three repetitions of each task were completed, and the best performed repetition was graded. Approximately five seconds of rest were provided between trials, one minute of rest between tests, and participants returned to the starting position between each trial (Okada et al., 2011). Participants were recorded by two video camcorders (Sony Electronics Inc., Tokyo, Japan), positioned anteriorly and laterally. Two qualified exercise scientists, trained and experienced with the FMS, analyzed participants live and later reviewed the video footage if required, and scored each participant individually. Movements were scored from 0–3. Scores of 3, 2, 1, and 0, represented, according to relevant criteria: ‘performed without compensation’, ‘performed with a single compensation’, ‘performed with multiple compensations or could not perform’, and ‘pain’, respectively (Cook et al., 2006a, 2006b; Frost et al., 2012). If there was any scoring discrepancy between the investigators, they reviewed the footage and discussed the result until a resolution was reached. This was done to minimize any discrepancies that may result between scorers (Shultz et al., 2013). Except for the deep squat and the trunk stability push-up, each side of the body was assessed within the movements, and all scores were considered in the analysis for this study.

### Modified Star Excursion Balance Test (mSEBT)

**Figure 1:**
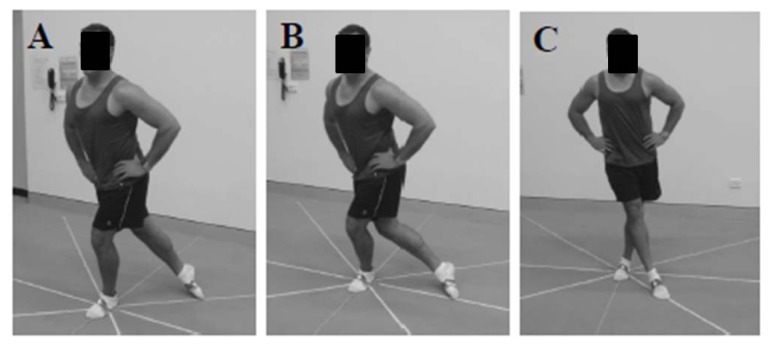
Modified Star Excursion Balance Test performance with a left stance leg and a right reach leg for the (A) posteromedial; (B) medial; and (C) anteromedial excursions

Dynamic balance was assessed by using the mSEBT through three excursions (posteromedial, medial, and anteromedial), which are shown in Figure 1. The testing grid consisted of 120-centimeter long tape measures taped to the laboratory floor. Each tape measure extended from an origin at 45º increments, measured by a goniometer. Participants stood on the center marker of the mSEBT, with the ankle malleoli aligned with lateral tape measures, which were visually assessed by the researcher. Participants then used their free leg to reach in the aforementioned order. With each attempt, the participant attempted to reach as far as possible along each line and make a light touch on the ground with the most distal part of the reaching leg. The participant then returned the reaching leg to a bilateral stance, without allowing this movement to affect overall balance. A researcher noted the distance after each attempt. Participants placed their hands on their hips during the mSEBT, and kept them there throughout all reach attempts. A trial was disregarded if the researcher felt the participant used the reaching leg for an extended period of support, removed the stance leg from the grid, removed their hands from their hips, or did not maintain balance. A minimum of three practice trials were used prior to data collection to familiarize participants to the movements required, and to serve as a warm-up. The order of the stance leg used during testing was randomized across participants. Reach distances were considered relative to leg length, and expressed as a percentage: *relative reach distance = reach distance/leg length x 100* (Gribble and Hertel, 2003; Lockie et al., in press).

### Statistical Analysis

All statistics were computed using the Statistics Package for Social Sciences Version 22.0 (IBM, Armonk, United States of America). Descriptive statistics (mean ± standard deviation) were used to profile each parameter. The Levene statistic determined homogeneity of variance of the data. Following established procedures (Frost and Cronin, 2011; Lockie et al., 2011; Lockie et al., 2013b; Spiteri et al., 2013), participants were ranked and split into high-, intermediate-, and low-performing dynamic stability groups according to two methods. The two ranking methods were the mean of reach distances when the right leg was used for the stance in the mSEBT, and the mean of reach distances when the left leg was used for the stance. As there is a tendency for dichotomized data to regress towards the mean, the participants ranked 14 and 28 for each dichotomization method were removed from the analysis, and groups of 13 participants each were established. This was done to ensure each group comprised participants of different dynamic stability levels. Thus, participants ranked 1–13 were in the high-performing group; participants ranked 15–27 were placed in the intermediate-performing group; and participants ranked 29–41 became the low-performing group. According to these groups, a one-way analysis of variance computed any significant (*p* < 0.05) differences between the selected individual screening exercises and mSEBT reach distances. Post hoc analysis was conducted for between-group pairwise comparisons using a Bonferroni adjustment for multiple comparisons.

Data was then pooled (n = 41) for a Pearson’s correlation analysis (*p* < 0.05) conducted between the deep squat, the left and right leg hurdle step, the in-line lunge, the active straight-leg raise, the trunk stability push-up, and the mSEBT scores. This analysis determined the relationships between performance in the individual screens, and dynamic stability as measured by functional reach distance. The strength of the correlation coefficient (r) was designated as per Hopkins (2009). An r value between 0 to 0.30, or 0 to −0.30, was considered small; 0.31 to 0.49, or −0.31 to −0.49, moderate; 0.50 to 0.69, or −0.50 to −0.69, large; 0.70 to 0.89, or −0.70 to −0.89, very large; and 0.90 to 1, or −0.90 to −1, near perfect for predicting relationships.

## Results

Table 1 displays the participants’ descriptive data and screening scores for each group when both the right (left leg reach), and left (right leg reach) legs were used for the mSEBT stance. No participant scored 0 for any of the screening exercises. There were no between-group differences for age (*p* = 0.47–1.00), body height (*p* = 1.00 for all between-group comparisons) or body mass (*p* = 1.00) for either grouping condition. There were also no significant differences in the deep squat (*p* = 1.00), the trunk stability push-up (*p* = 0.90–1.00), or the hurdle step (*p* = 0.06–1.00), the in-line lunge (*p* = 0.11–1.00) and the active-straight leg raise (*p* = 0.08–1.00) for either leg, for each mSEBT stance group dichotomization.

Table 2 shows the mSEBT reach distances when the right and left stance leg mSEBT totals were used to delineate the groups. When both legs were used for the stance, the high-performing group was significantly (*p* ≤ 0.02) better than the low-performing group for all excursion measures, and significantly (*p* ≤ 0.01) superior in all but the anteromedial excursions when compared to the intermediate group. The intermediate-performing group performed significantly (*p* ≤ 0.01) better in all but the anteromedial excursions when compared to the low-performing group.

The correlations between mSEBT and FMS scores are shown in Table 3. The trunk stability push-up had a moderate positive relationship (*p* = 0.02) with the right stance leg posteromedial excursion, and moderate negative relationships (*p* = 0.04) with the right and left stance leg anteromedial excursions. The left leg in-line lunge had a moderate positive relationship (*p* < 0.01) with the right-leg posteromedial excursion when the left leg was used for the stance. There were no other significant relationships between the mSEBT and the screen scores.

## Discussion

To the authors’ knowledge, this is the first study to investigate relationships between specific FMS exercises and dynamic stability as measured by the mSEBT in team sport athletes. The results of this study generally showed that there were no relationships between the screens and dynamic stability as measured by the mSEBT. When participants were dichotomized into high-, intermediate-, and low-performing dynamic stability groups, there were no significant differences in performance of any screening exercise (Table 1). Furthermore, only four correlations between the mSEBT and FMS exercises were significant, and two of these significant relationships suggested that a poorer score in the screen (the trunk-stability push-up) related to a further anteromedial excursion (Table 3). This was counter to the studies’ hypothesis, and occurred even through the analyzed screens are said to challenge dynamic stability within a functional movement (Cook et al., 2006a, 2006b). The results from this study appear to support the research that found the FMS to have limited to no relationship to athletic performance (Lockie et al., 2015; Okada et al., 2011; Parchmann and McBride, 2011).

If the deep squat, the hurdle step, the inline lunge, the active straight-leg raise, and the trunk stability push-up had provided an indication of dynamic stability, it would have been assumed team sport athletes who exhibit better dynamic stability would also perform better in these screens. However, this was not the case. There were no differences between the groups comprising participants with high, intermediate, or low dynamic stability capabilities (Table 1). The results from this study imply that the qualities measured from functional lower-limb reaching and the mSEBT, which are valid tests of dynamic stability (Hertel et al., 2006; Olmsted et al., 2002; Robinson and Gribble, 2008), appear to be relatively disparate from that assessed in the FMS by the hurdle step and the in-line lunge.

These findings were also reinforced by the results from the correlation analyses (Table 3). There were only two significant positive relationships between the screens and the mSEBT (the trunk stability push-up and the in-line lunge with posteromedial excursions). This was despite previous research finding significant correlations between FMS exercises and a different measure of dynamic stability in the Y-balance test in soldiers (Teyhen et al., 2014). Nevertheless, even though there were significant relationships found by Teyhen et al. (2014) with screens including the deep squat, the hurdle step, and the in-line lunge, using parameters set by Hopkins (2009), the strength of these correlations documented was still relatively weak. Taken together with the between-group analysis from this study, any suggestion that exercises from the FMS can provide some type of measure of dynamic stability appear to be questionable. This is an important concern for strength and conditioning coaches who may use a screening tool such as the FMS, and what they can surmise about the results they attain from their athletes. Coaches would be better served to use valid assessments such as the mSEBT, which is also reinforced by findings from the current research.

When either leg was used for the stance, the mSEBT distinguished team sport athletes with different dynamic stability capabilities (Table 2). This supports the work of Hertel et al. (2006), who stated that the posteromedial, medial, and anteromedial excursions best represented dynamic stability measured by reach distances. Furthermore, the mSEBT and its variations have been shown to relate to multidirectional speed (Lockie et al., in press), and can be improved through specific training (Filipa et al., 2010; Lockie et al., 2014b; Valovich McLeod et al., 2009). Therefore, strength and conditioning coaches could use the mSEBT to assess dynamic stability in their athletes, with the knowledge that it is applicable to team sport athletes, will delineate between athletes of different dynamic stability capabilities, and can be enhanced through appropriate training.

There were certain limitations associated with this study. Although it is a valid test (Hertel et al., 2006), the mSEBT was the only measure of dynamic stability utilized. Indeed, there are several different dynamic stability assessments used by practitioners in the field (Dallinga et al., 2012), including the Y-balance (Teyhen et al., 2014) or hop-and-balance (Myer et al., 2006) tests. The FMS could potentially relate to these alternate assessments. Males and females can demonstrate different movement biomechanics during certain actions (McLean et al., 2004), and the combined gender approach may have influenced the study results. However, this approach had been used in previous FMS (Okada et al., 2011; Parchmann and McBride, 2011; Teyhen et al., 2014) and sports technique (Eikenberry et al., 2008; Guissard et al., 1992; Lockie et al., 2012; Spiteri et al., 2013) research, and thus was viewed as appropriate. Correlation analyses do not establish cause-and-effect between variables, in that factors such as the participants’ physical characteristics, flexibility, technique, and strength can influence the statistical models that are derived (Brughelli et al., 2008). Lastly, the use of other methods of analysis, such as electromyography or force plates, would also be useful to elucidate any technical similarities between the characteristics of the FMS exercises and the mSEBT. Electromyography has been used in the literature to demonstrate leg muscle activation patterns during SEBT excursions (Earl and Hertel, 2001; Norris and Trudelle-Jackson, 2011), while a force plate has been used to track postural sway and the center of pressure pattern during a stability task (Brown and Mynark, 2007; Gribble et al., 2007). Nonetheless, this research is still valuable for strength and conditioning coaches, as the findings demonstrate that unlike the mSEBT, FMS exercises such as the deep squat, the hurdle step, the in-line lunge, the active straight-leg raise, and the trunk stability push-up have a limited capacity to indicate dynamic stability in team sport athletes.

The results of the current study document the limited application of FMS exercises to provide some indication of dynamic stability in team sport athletes. The FMS may have value in monitoring movement deficits that could increase the risk of injury in athletes, although this is still to be confirmed. However, as for previous research (Lockie et al., 2013a; Lockie et al., 2015; Okada et al., 2011; Parchmann and McBride, 2011), the screens have restricted application to athletic performance. In contrast, the mSEBT can be used to delineate between team sport athletes of different dynamic stability capabilities. Strength and conditioning coaches who use the FMS as a measure of dynamic stability should be aware that the attained scores may not provide an accurate assessment of this capacity in their athletes. Thus, an assessment such as the mSEBT should also be included in an athlete’s testing protocol. Coaches who use the mSEBT can be confident that they will be utilizing an assessment that will provide a valid assessment of dynamic stability in team sport athletes, which may also provide useful data for training progress or team selection.

## Figures and Tables

**Table 1:** Descriptive statistics (age = year; body height = meters; body mass = kilograms) and screening scores (deep squat; hurdle step: HS; in-line lunge: ILL; active-straight-leg raise: ASLR; trunk stability push-up: TSPU) for high-, intermediate-, and low-performing groups as defined by mean reach distance in the modified Star Excursion Balance Test for each leg by high-, intermediate-, and low-performing recreational team sport athletes. Reach performance was defined from both when the right leg (left reach leg) and left leg (right reach leg) were used for the stance. Screening scores are out of 3

	High (n = 13)	Intermediate (n = 13)	Low (n = 13)
*Groups defined by Right Stance Leg – Left Reach Leg Total Score*			
Age	23.54 ± 4.74	22.69 ± 3.86	21.31 ± 3.01
Body Height	1.77 ± 0.10	1.76 ± 0.08	1.76 ± 0.09
Body Mass	72.94 ± 11.47	76.98 ± 9.63	76.69 ± 16.55
Deep Squat	1.69 ± 0.86	1.62 ± 0.65	1.62 ± 0.65
HS Left	1.85 ± 0.69	1.38 ± 0.65	1.38 ± 0.77
HS Right	2.08 ± 0.76	1.38 ± 0.65	1.62 ± 0.77
ILL Left	2.62 ± 0.51	2.08 ± 0.76	2.15 ± 0.90
ILL Right	2.54 ± 0.66	1.92 ± 0.76	2.23 ± 0.73
ASLR Left	2.62 ± 0.65	1.92 ± 0.86	2.38 ± 0.77
ASLR Right	2.54 ± 0.66	2.15 ± 0.90	2.31 ± 0.86
TSPU	2.23 ± 0.83	2.08 ± 0.76	1.92 ± 0.64
*Groups defined by Left Stance Leg – Right Reach Leg Total Score*			
Age	23.46 ± 4.70	23.62 ± 4.65	21.62 ± 3.12
Body Height	1.75 ± 0.09	1.77 ± 0.07	1.76 ± 0.08
Body Mass	75.94 ± 13.56	75.36 ± 12.40	76.46 ± 13.09
Deep Squat	1.77 ± 0.93	1.62 ± 0.51	1.77 ± 0.73
HS Left	1.77 ± 0.83	1.46 ± 0.66	1.38 ± 0.51
HS Right	2.00 ± 0.82	1.54 ± 0.66	1.54 ± 0.78
ILL Left	2.54 ± 0.52	2.31 ± 0.75	2.15 ± 0.90
ILL Right	2.46 ± 0.66	2.08 ± 0.86	2.23 ± 0.73
ASLR Left	2.54 ± 0.66	2.08 ± 0.95	2.38 ± 0.77
ASLR Right	2.46 ± 0.66	2.23 ± 0.93	2.31 ± 0.86
TSPU	2.31 ± 0.86	2.00 ± 0.71	2.15 ± 0.69

**Table 2:** Modified Star Excursion Balance Test (mSEBT) performance for high-, intermediate-, and low-performing groups as defined by mean reach distance in the mSEBT for each leg by high-, intermediate-, and low-performing male and female recreational team sport athletes. Reach performance was defined from both when the right leg (left reach leg) and left leg (right reach leg) were used for the stance. Excursion distances were defined as a percentage of leg length.

	High (n = 13)	Intermediate (n = 13)	Low (n = 13)
*Groups defined by Right Stance Leg – Left Reach Leg Total Score*			
Posteromedial	96.35 ± 4.83	87.28 ± 4.44*	76.82 ± 7.45*†
Medial	88.48 ± 9.06	79.41 ± 3.32*	68.92 ± 6.89*†
Anteromedial	79.01 ± 4.84	76.52 ± 5.44	71.74 ± 6.90*
Mean Reach	87.95 ± 3.65	81.07 ± 1.15*	72.49 ± 4.21*†
*Groups defined by Left Stance Leg – Right Reach Leg Total Score*			
Posteromedial	94.49 ± 3.85	84.23 ± 5.32*	76.84 ± 4.65*†
Medial	89.56 ± 5.67	78.00 ± 5.17*	66.53 ± 8.02*†
Anteromedial	78.54 ± 6.20	73.68 ± 5.19	71.42 ± 7.33*
Mean Reach	87.53 ± 3.41	78.64 ± 1.49*	71.60 ± 2.62*†

* Significantly (p < 0.05) less than the high-performing group.

† Significantly (p < 0.05) less than the intermediate-performing group.

**Table 3:** Correlations between reach distances in the modified Star Excursion Balance Test when the right (left leg reach) and left (right leg reach) legs were used for the stance and performance in the deep squat, the left- and right-leg hurdle step, the left- and right-leg in-line lunge, the left- and right-leg active straight-leg raise, and the trunk stability push-up in recreational team sport athletes (n = 41).

	Posteromedial	Medial	Anteromedial	Mean Reach
*Right Stance Leg – Left Reach Leg Excursions*				
Deep Squat	0.02	−0.10	0.04	−0.02
Hurdle Step Left	0.23	0.26	0.27	0.31
Hurdle Step Right	0.29	0.24	0.14	0.29
In-line Lunge Left	0.27	0.27	−0.11	0.22
In-line Lunge Right	0.20	0.14	−0.17	0.11
Active Straight-Leg Raise Left	0.10	0.18	−0.03	0.13
Active Straight-Leg Raise Right	0.02	0.14	<0.01	0.08
Trunk Stability Push-Up	0.37*	0.13	−0.33*	0.14
*Left Stance Leg – Right Reach Leg Excursions*				
Deep Squat	−0.05	0.01	−0.05	−0.03
Hurdle Step Left	0.20	0.25	0.24	0.29
Hurdle Step Right	0.16	0.25	0.12	0.24
In-line Lunge Left	0.46*	0.30	−0.25	0.27
In-line Lunge Right	0.28	0.17	−0.20	0.15
Active Straight-Leg Raise Left	0.14	0.18	−0.03	0.14
Active Straight-Leg Raise Right	0.07	0.18	−0.03	0.12
Trunk Stability Push-Up	0.26	0.15	−0.32*	0.08

* Significant (p < 0.05) relationship between the two variables.
